# New-Onset Graves’ Disease Presenting As Thyro-Pericarditis

**DOI:** 10.7759/cureus.65301

**Published:** 2024-07-24

**Authors:** Ji-Cheng Hsieh, Spencer Weintraub, Karim Diab, Ally W Wang, Robert Copeland-Halperin

**Affiliations:** 1 Internal Medicine, Northwell Cardiovascular Institute, New Hyde Park, USA; 2 Cardiology, Northwell Cardiovascular Institute, New Hyde Park, USA; 3 Endocrinology, Northwell Cardiovascular Institute, New Hyde Park, USA

**Keywords:** thyrotoxicosis, pericarditis, thyrotoxic pericarditis, myopericarditis, thyro-pericarditis, graves´disease

## Abstract

Acute perimyocarditis is commonly preceded by viral illness and presents with non-specific complaints that can be a manifestation of serious cardiac complications such as arrhythmias and heart failure. While pericarditis is a known complication of thyrotoxicosis, termed “thyrotoxic pericarditis,” concomitant new-onset perimyocarditis and Graves’ disease, termed “thyro-pericarditis,” has been reported. We present a case of thyro-pericarditis as the initial presentation of undiagnosed and untreated Graves’ disease co-occurring with recent Coxsackievirus A and B infection.

A 27-year-old male with a family history of undifferentiated hyperthyroidism presented with acute pleuritic chest pain and shortness of breath. Laboratory testing showed elevated cardiac troponin I with ST elevations and PR depressions on initial ECG. Left heart catheterization was normal, but transthoracic echocardiogram showed right ventricular systolic dysfunction and enlargement. Cardiac MRI demonstrated diffuse pericardial enhancement suggesting pericarditis. Thyroid function testing and thyroid ultrasound suggested auto-immune thyrotoxicosis. Serology noted abnormal Coxsackievirus A and B IgG antibody titers, suggesting prior infection. The patient was treated with colchicine, ibuprofen, methimazole, and metoprolol, with resolution of symptoms. Thyro-pericarditis is a rare concomitant presentation of both Graves’ disease and myopericarditis, and it remains unknown whether there is an increased risk of adverse cardiac outcomes.

## Introduction

Acute myopericarditis typically presents with non-specific complaints of chest pain, palpitations, dyspnea, and exercise intolerance [1]. While most patients recover, myopericarditis can be complicated by atrioventricular block, ventricular arrhythmia, heart failure with dilated cardiomyopathy, and sudden cardiac death [1]. The majority of cases of acute myopericarditis in developed countries are from viral infections (group B Coxsackieviruses, Epstein-Barr virus, human herpes virus 6, and parvovirus B19). However, bacterial (*Corynebacterium diptheriae*), parasitic (*Trypanosoma cruzi*), and autoimmune causes are common in developing countries [1,2]. HIV and viral hepatitis can also lead to myopericarditis [3,4]. Timely evaluation to rule out ischemic or valvular heart disease and treatment of myopericarditis are critical to preventing complications. While histological diagnosis with endomyocardial biopsy is definitive, clinical diagnosis, combined with laboratory testing and imaging studies such as electrocardiogram (ECG), echocardiography, and cardiac MRI, can assist in establishing the diagnosis [1]. Myopericarditis can also co-occur with Graves’ disease, a diagnostic entity termed “thyro-pericarditis,” where it is critical to manage concomitant Graves’ disease with thyroid suppression via methimazole, beta blockers, or, in rare cases, glucocorticoids. Graves’ disease similarly can be triggered or exacerbated by a viral infection. It remains unknown whether a shared process leads to both myopericarditis and autoimmune thyroid disease or if one leads to the other. Additionally, it remains unknown if concomitant presentation leads to greater risk of adverse cardiac outcomes. It remains essential that providers be aware that the possible presentation of pericarditis could be due to a potential underlying thyroid condition. We present a case of a young man with acute myopericarditis in the setting of undiagnosed Graves’ disease. Our case represents the unique first presentation of myopericarditis as the initial presentation for Graves’ disease and is complicated by a recent upper respiratory illness at the time of his myopericarditis making the causal relationship challenging.

This article was previously presented as a meeting abstract at the 2024 ACC-New York “Shark Tank Challenge” and “Young Investigator’s Award Competition” on October 21, 2023, as well as 2023 Society of Hospital Medicine Converge on March 28, 2023.

## Case presentation

**Figure 1:**
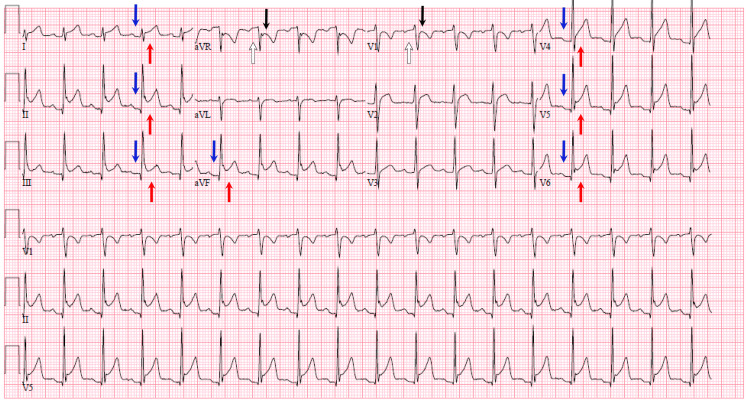
Diffuse ST elevations (red arrows) and PR depressions (blue arrows), with reciprocal ST depressions (black arrows) and PR elevations (white arrows) in aVR and V1 on initial ECG, suggestive of pericarditis.

A 27-year-old male with no past medical or surgical history presented with acute onset pleuritic chest pain and shortness of breath. The patient reported subjective fevers and myalgia for one day prior to presentation. He reported recent nasal congestion, runny nose, and cough one week earlier, which had resolved. Over the past year, the patient reported having 25 kilograms of unintentional weight loss, heat intolerance, and episodes of diaphoresis. There was no history of new medications or recent vaccinations. He immigrated from Thailand to the United States one year prior to presentation, but there was no recent international travel. The patient’s father had hyperthyroidism, but the patient himself denied any personal diagnosis of a thyroid disorder. On examination, he was afebrile, heart rate was 110 beats/min (regular), and blood pressure was normal. The appearance of the eyes, face, hair, and skin were normal, and examination of the neck revealed a homogenously enlarged thyroid gland, with no discrete nodules or goiter. The patient reported no tenderness to superficial and deep palpation of the neck. Auscultation of the chest revealed normal heart sounds and no friction rub. The abdomen was benign. The extremities were warm, and there was no rash or edema. Infectious testing (Table 1) was negative for HIV-1 p24 antigen and HIV-1/HIV-2 IgG/IgM antibodies on combination immunoassay. Testing was also negative for hepatitis B core IgM antibody, hepatitis B surface antigen, hepatitis A IgM antibody, and hepatitis C IgG/IgM antibodies. COVID-19 PCR was negative. However, Coxsackievirus type A and B IgG antibody titers were positive for prior infection. Coxsackievirus type A7, A9, and A16 IgG antibody titers were 1:400, and A24 titer was 1:800 (normal <1:100). Coxsackievirus type B2, B3, B4, and B6 IgG antibody titers were 1:16, and B5 titer was 1:32 (normal <1:8). Initial laboratory testing showed cardiac troponin I 5,292.7 ng/L (upper limit normal [ULN] < 76 ng/L), CK-MB 77.5 ng/mL (ULN <6.7 ng/mL), and N terminal pro-brain natriuretic peptide concentration 314 pg/mL (ULN <300 pg/mL). ECG showed sinus rhythm with diffuse ST elevations and PR depressions, with reciprocal ST depressions and PR elevations in aVR and V1 (Figure 1).

**Table 1:** Viral infectious testing on admission

Viral Testing (Normal Range)	On Admission
Hepatitis B core IgM antibody	Nonreactive
Hepatitis B surface antigen	Nonreactive
Hepatitis A IgM antibody	Nonreactive
Hepatitis C virus signal-to-cutoff ratio (0.00-0.99)	0.24
Coxsackie type B1 antibodies (<1:8)	1:8
Coxsackie type B2 antibodies (<1:8)	1:16
Coxsackie type B3 antibodies (<1:8)	1:16
Coxsackie type B4 antibodies (<1:8)	1:16
Coxsackie type B5 antibodies (<1:8)	1:32
Coxsackie type B6 antibodies (<1:8)	1:16
Coxsackie type A7 (<1:100)	1:400
Coxsackie type A9 (<1:100)	1:400
Coxsackie type A16 (<1:100)	1:400
Coxsackie type A24 (<1:100)	1:800
HIV-1 p24 antigen	Nonreactive
HIV-1/2 antibody	Nonreactive
COVID-19 nucleic acid amplification test	Nonreactive

**Figure 2:**
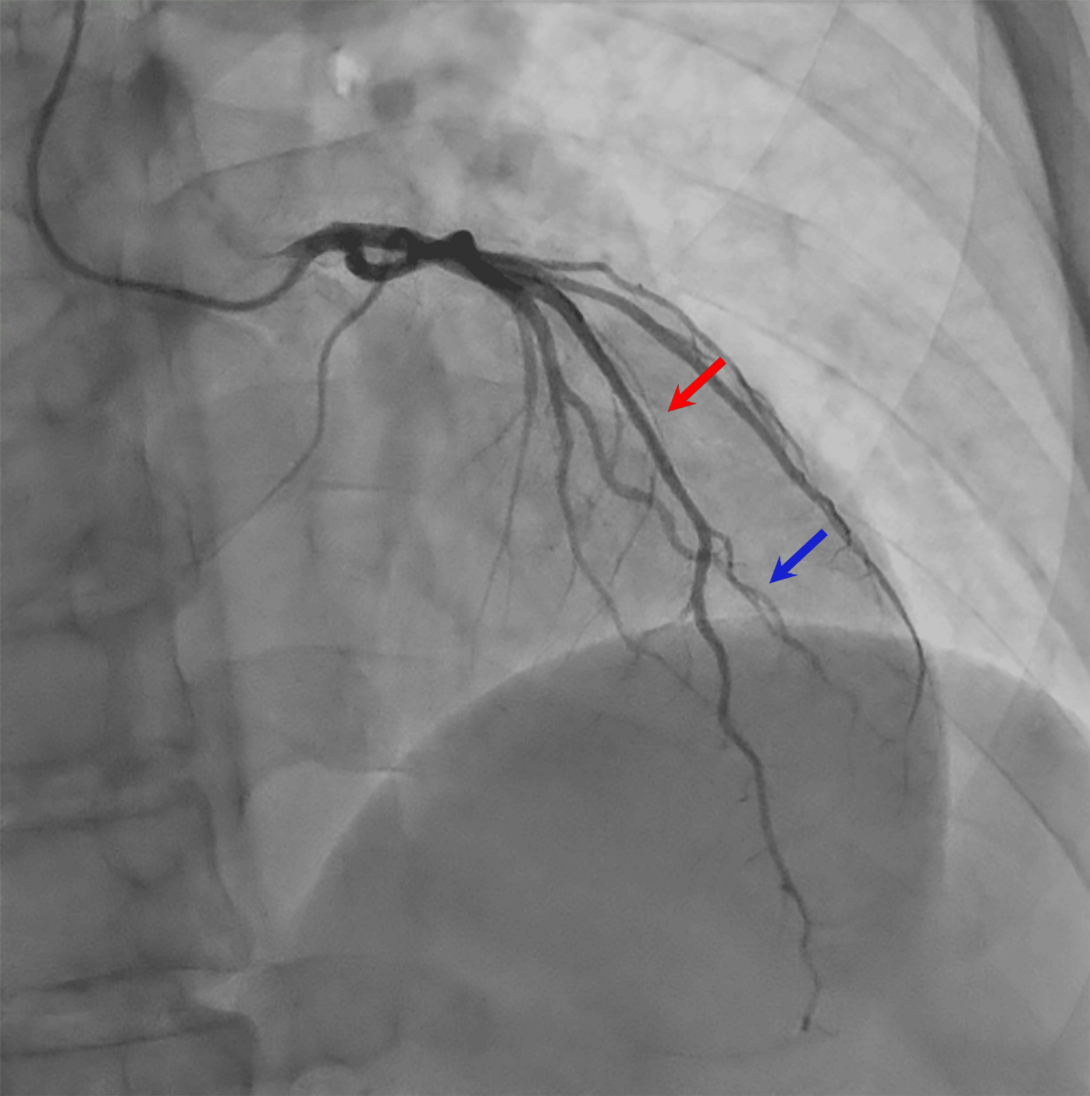
Right anterior oblique cranial view during coronary angiography, demonstrating no stenoses or abnormalities of the left anterior descending (red arrow) and diagonal branch (blue arrow).

**Figure 3:**
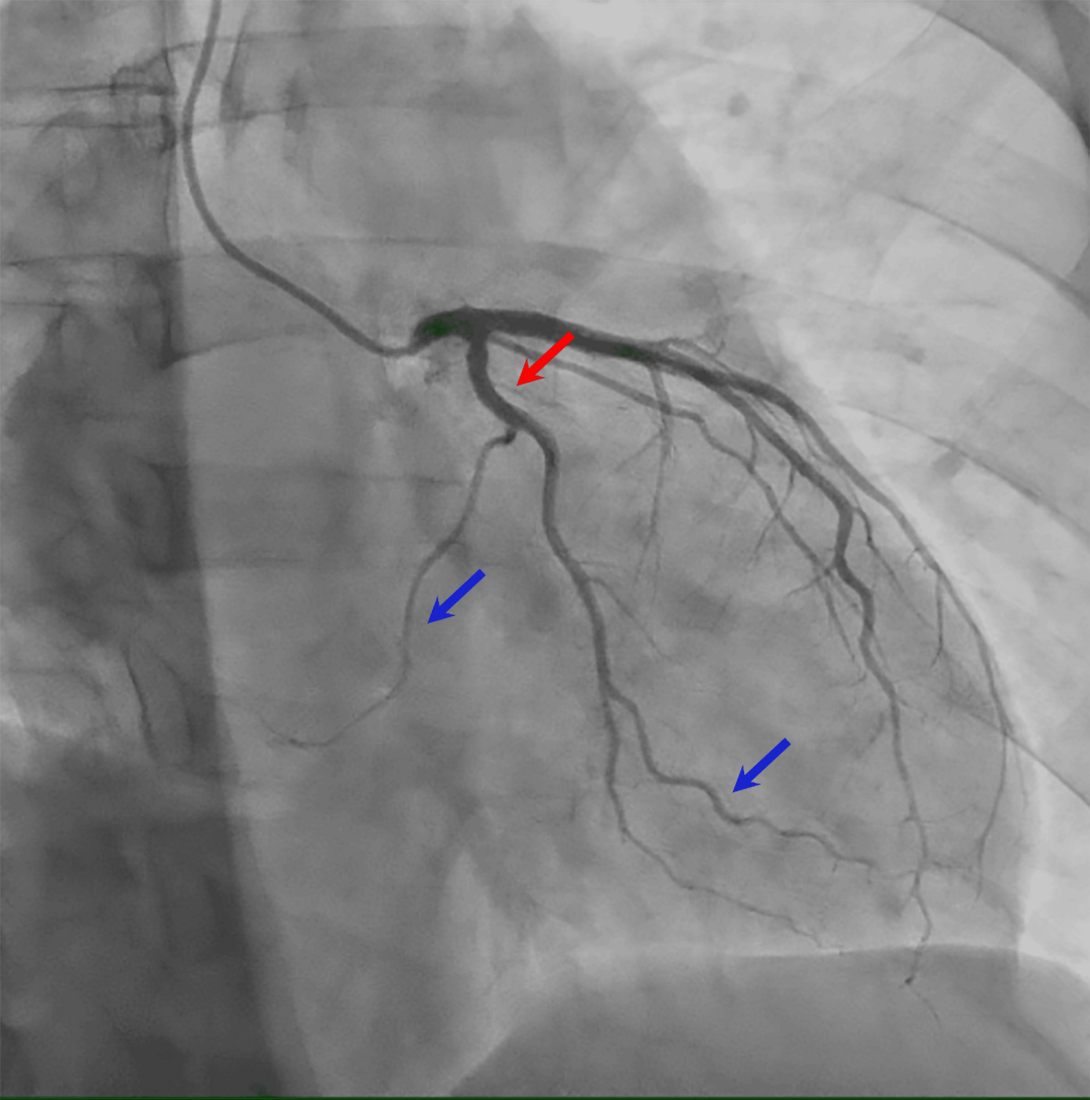
Right anterior oblique caudal view during coronary angiography, demonstrating no stenoses or abnormalities of the left circumflex (red arrow) and left marginal arteries (blue arrows).

**Figure 4:**
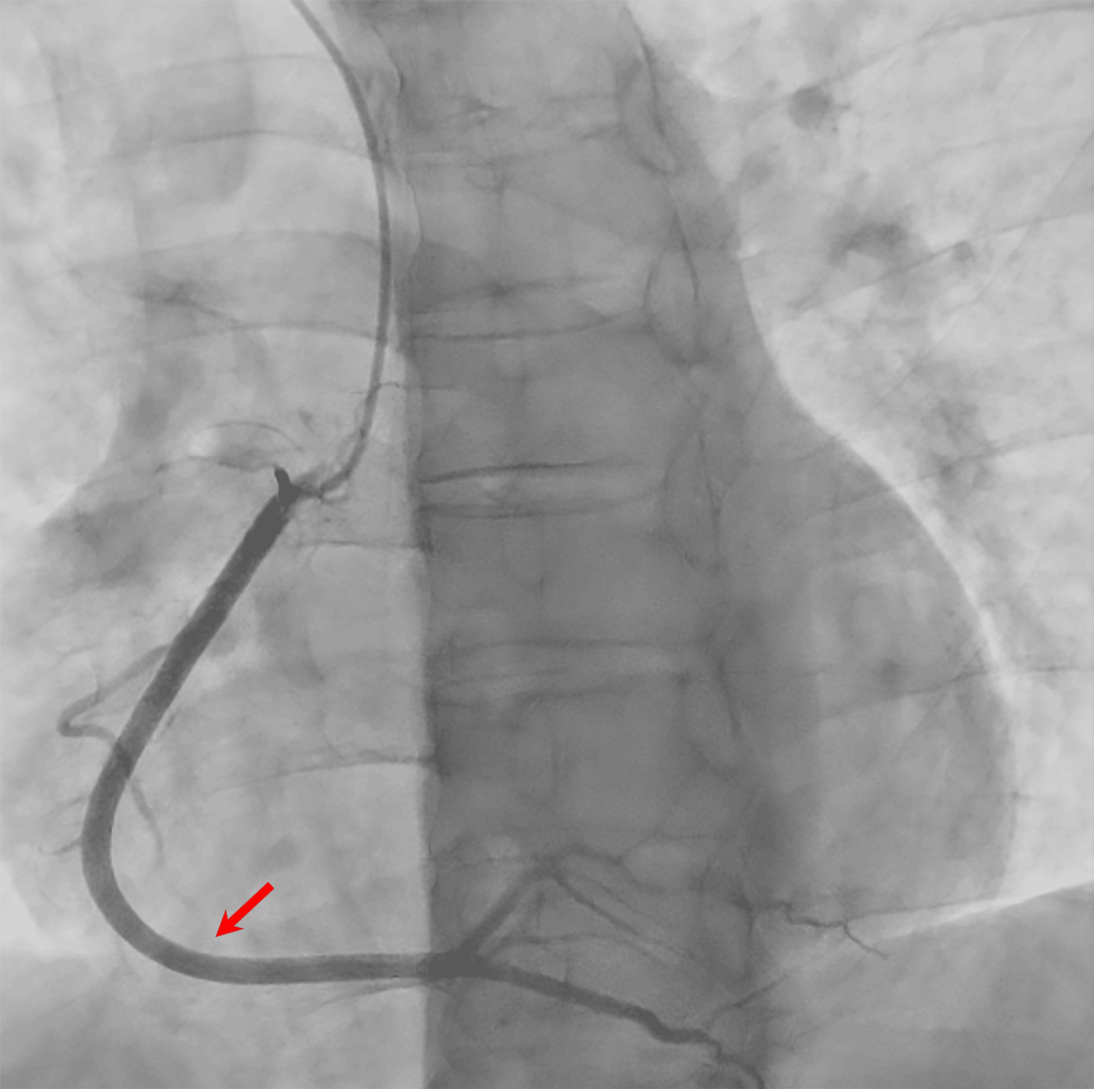
Right coronary artery view during coronary angiography, demonstrating no stenoses or abnormalities of the right coronary artery (red arrow).

**Figure 5:**
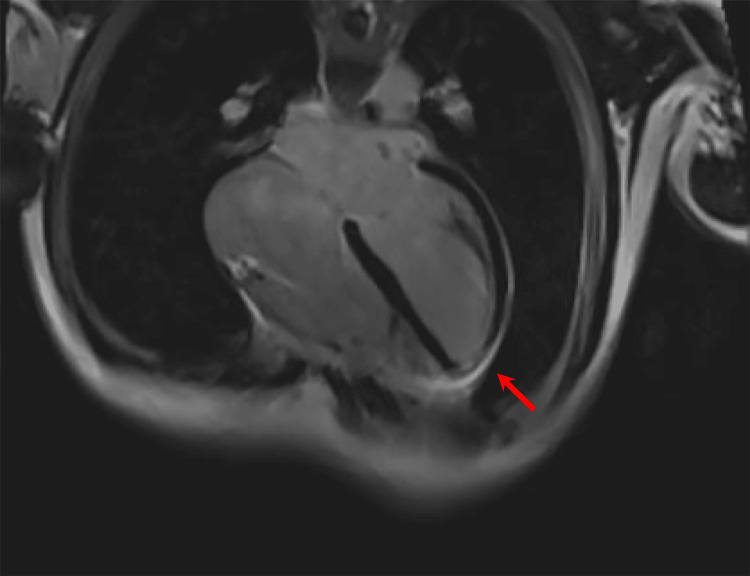
Axillary cardiac MRI view demonstrating diffuse pericardial enhancement (red arrow) consistent with a diagnosis of pericarditis.

**Figure 6:**
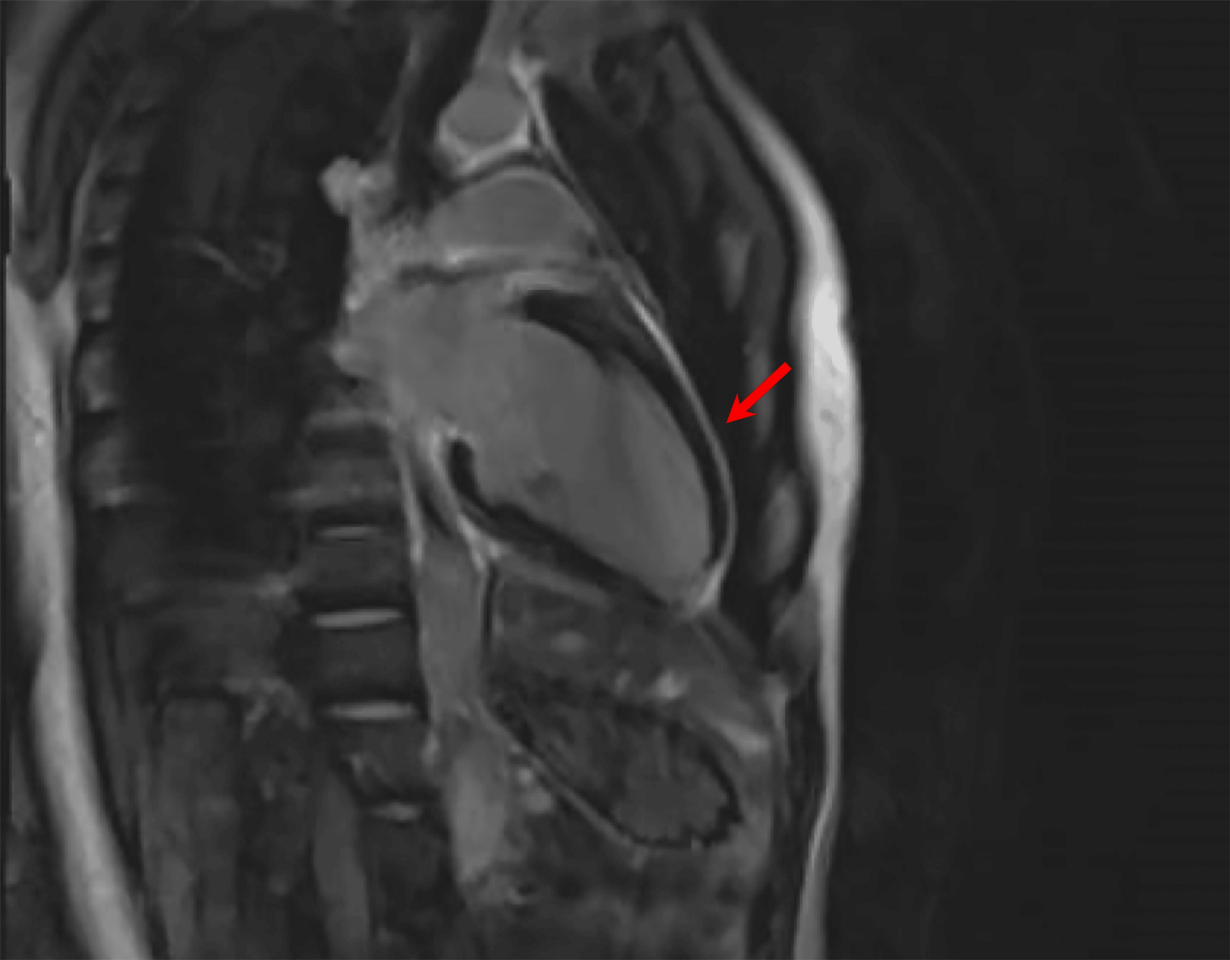
Sagittal cardiac MRI view demonstrating diffuse pericardial enhancement (red arrow) consistent with a diagnosis of pericarditis.

Left heart catheterization showed normal anatomy and no obstructive epicardial coronary artery disease (Figures 2-4). Transthoracic echocardiogram showed right ventricular (RV) enlargement with abnormal septal motion and decreased systolic function, suggestive of RV overload. Left ventricular ejection fraction (LVEF) was 64%. No pericardial effusion was noted. Quantitative d-dimer was normal at <150 (ULN <229 ng/mL), suggesting against pulmonary embolism. Cardiac MRI revealed diffuse pericardial enhancement without pericardial thickening or effusion (Figures 5, 6). There was no evidence of myocardial scar or edema. LVEF was 56%, with normal left atrial (LA) and left ventricular (LV) size. RV ejection fraction was 51%, with enlarged RV size. The patient met the criteria for acute pericarditis with clinical symptoms of pleuritic chest pain and ECG findings of diffuse ST elevations and PR depressions, with reciprocal change in aVR and V1, consistent with the first of four ECG stages seen in pericarditis [5,6].

Basic thyroid testing for suspected hyperthyroidism was ordered according to the 2016 American Thyroid Association Guidelines (Table 2) [7]. Basic thyroid testing was notable for thyroid-stimulating hormone (TSH) <0.007 uIU/mL (normal range: 0.3-3.7 uIU/mL), total T3 208 ng/dL (normal range: 80-200 ng/dL), and free T4 4.6 ng/dL (normal range: 0.9-1.8 ng/dL). The patient also had elevated C-reactive protein of 67 mg/L (normal range: 0-4 mg/L) and Westergren sedimentation rate of 46 mm/hr (normal range: 0-15 mm/hr). Additional testing was notable for elevated anti-TSH receptor antibodies (TRAb) at 14.70 IU/L (normal range: 0.00-1.75 IU/L), elevated thyroid-stimulating immunoglobulin at 2.01 IU/L (normal range: 0.00-0.55 IU/L), and normal anti-thyroperoxidase antibodies at 16.7 IU/mL (normal range: <34.9 IU/mL). Thyroid ultrasound revealed diffuse hyperemia with high vascularity, typically seen in Graves’ disease.

**Table 2:** Thyroid testing on admission and at eight-month follow-up. TSH, thyroid-stimulating hormone

Thyroid Laboratory Testing (Normal Range)	On Admission	At 8-Month Follow-up
TSH (0.3-3.7 uIU/mL)	<0.007 uIU/mL	<0.01 uIU/mL
Total T3 (80-200 ng/dL)	208 ng/dL	Not performed
Free T3 (2.00-4.40 pg/mL)	Not performed	12.30 pg/mL
Free T4 (0.9-1.8 ng/dL)	4.6 ng/dL	4.9 ng/dL
TSH receptor antibodies (0.00-1.75 IU/L)	14.70 IU/L	Not performed
Thyroid-stimulating immunoglobulin (0.00-0.55 IU/L)	2.01 IU/L	Not performed
Thyroperoxidase antibody (<34.9 IU/mL)	16.7 IU/mL	85.0 IU/mL
Thyroglobulin antibody (<20.0 IU/mL)	Not performed	<20.0 IU/mL

**Figure 7:**
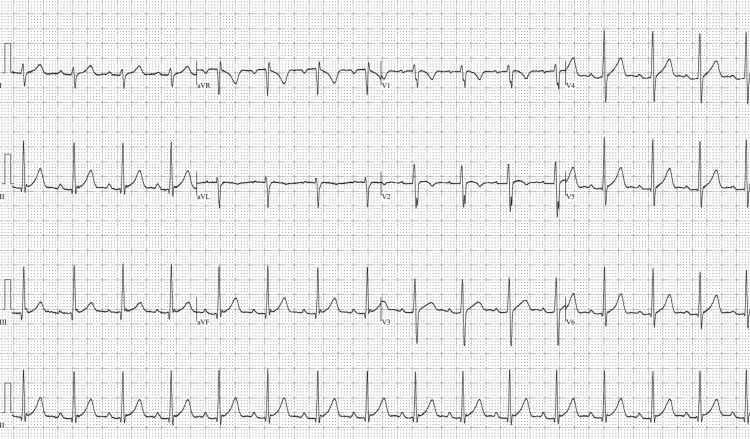
Repeat ECG eight months after discharge showing resolution of ST elevations and PR depressions.

The patient was treated with colchicine, ibuprofen, methimazole, and metoprolol, with resolution of his chest pain and tachycardia. He received methimazole for three months due to poor initial follow-up. Follow-up thyroid tests eight months after discharge (Table 2) showed TSH <0.01 uIU/mL, free T3 12.30 pg/mL, free T4 4.9 ng/dL, thyroperoxidase antibody 85.0 IU/mL, and thyroglobulin antibody <20.0 IU/mL, and C-reactive protein was normal (<3 mg/L). A repeat ECG eight months after discharge (Figure 7) showed resolution of diffuse PR depressions and ST elevations seen in initial ECG during hospitalization. Repeat echocardiogram noted an LVEF of 65% and normal RV systolic function.

## Discussion

We describe a case of first presentation Graves’ disease and myopericarditis in the setting of Coxsackievirus A and B infection, which to our knowledge has not been previously reported. Viral infection is a shared common cause of Graves’ disease, thyroiditis, and myopericarditis, and there have been reports documenting simultaneous presentation of both Graves’ disease and pericarditis, termed “thyro-pericarditis” [8,9]. This patient’s laboratory findings of overt hyperthyroidism, with low TSH, elevated total T3 and free T4, and elevated TRAb, suggested new onset Graves’ disease. Hyperemia, with increased vascularity, on thyroid ultrasound also supported this diagnosis. Competing causes of thyrotoxicosis such as subacute thyroiditis were considered but deemed less likely, based on the patient's history of significant weight loss, heat intolerance, and diaphoresis over the prior year, preceding and therefore unrelated to the more recent upper respiratory infection (URI) and the total absence of tenderness on palpation of the patient’s neck. However, elevated TRAb has been seen rarely in subacute thyroiditis, and, therefore, subacute thyroiditis could not be completely excluded in the setting of elevated global inflammatory markers [10]. Due to the acuity of the patient’s illness warranting treatment, we opted to treat this patient with a working diagnosis of Graves’ disease. Radioactive iodine uptake scan (RAIU) would have definitely ruled out subacute thyroiditis, but it was not available in the inpatient setting at our institution. An additional diagnostic limitation was that total T4 was not measured during the index hospitalization, prohibiting the calculation of the T3/T4 ratio, which could have further substantiated the diagnosis of Graves’ disease. Although it remains possible as an underlying cause of the patient’s presentation, it is less likely, given the clinical history and physical examination findings, that this patient had superimposed thyroiditis from the recent Coxsackievirus infection. Additionally, while the patient’s presentation met the criteria for acute pericarditis and was supported by limited involvement of the myocardium on cardiac MRI, biomarkers of cardiomyocyte injury suggested myocardial involvement.

Some reports postulate a distinct causative relationship and shared underlying autoimmune process between Graves’ disease and pericarditis [11], while other reports suggest that pericarditis develops from thymic hyperplasia secondary to Graves’ disease, supported by cases with no identified viral precipitant [12,13]. Considering the patient’s family history, he was likely genetically predisposed to developing autoimmune disease, and the patient’s report of clinical symptoms of thyrotoxicosis over the year before presentation suggests underlying undiagnosed thyrotoxicosis more likely due to Graves’ disease. While it is entirely possible that the patient’s Coxsackievirus type A and B infection may have triggered an episode of thyroiditis, it remains more likely that acute infection exacerbated the patient’s underlying untreated Graves’ disease and led to the patient’s presentation with thyro-pericarditis. While more definitive testing such as RAIU would have provided greater diagnostic insight, the unavailability of the test and acuity of the patient’s illness required the team to rely more on clinical and basic lab testing to establish a working diagnosis and treatment plan.

The interaction between autoimmune thyroid disease and myopericarditis is complex, and it is unknown whether concomitant Graves’ disease and myopericarditis further increases the risk of adverse cardiac events, given the risk of thyrotoxic cardiomyopathy in uncontrolled thyrotoxicosis. This is especially pertinent as the patient was asymptomatic on follow-up. However, auto-antibodies to thyroperoxidase persisted.

## Conclusions

It remains unclear whether there is a causative relationship between Graves’ disease and myopericarditis or if both are driven by a shared autoimmune process, such as viral infection. We describe a case of thyro-pericarditis at the first presentation of Graves’ disease, likely triggered by Coxsackievirus type A and B infection. Our case highlights the importance of a detailed history to assess for symptoms of hyperthyroidism, a thorough thyroid examination, comprehensive thyroid testing, and the utility of thyroid ultrasound. Physicians should recognize the existence of a relationship between pericarditis and Graves’ disease and consider thyroid disease in patients presenting with pericarditis as this has the potential to impact treatment and outcomes.
